# Trends in laboratory testing for diabetes in Ontario, Canada 1995–2005: A population-based study

**DOI:** 10.1186/1472-6963-9-41

**Published:** 2009-02-27

**Authors:** Sarah E Wilson, Lorraine L Lipscombe, Laura C Rosella, Douglas G Manuel

**Affiliations:** 1 Institute for Clinical Evaluative Sciences, 2075 Bayview Avenue, Toronto, Ontario, M4N 3M5, Canada; 2 Dalla Lana School of Public Health, University of Toronto, 155 College Street, Toronto, Ontario, M5T 3M7, Canada; 3 Women's College Hospital, 76 Grenville Street, Toronto, Ontario, M5S 1B2, Canada; 4 Department of Medicine, University of Toronto, Toronto, Ontario, Canada

## Abstract

**Background:**

There are concerns that testing for type 2 diabetes is low and many people with diabetes are not diagnosed. We sought to describe the rates of diabetes-related lab testing in Ontario from 1995–2005, among adults without diabetes, and to explore the extent to which the Canadian clinical practice guidelines for screening are being followed.

**Methods:**

Descriptive counts of outpatient diabetes laboratory tests performed within Ontario's publicly funded, provincial health insurance program were recorded. The study population was Ontario residents, 20 years and older from 1995 to 2005 (9.3 million people in 2005). The Ontario Diabetes Database, a cumulative registry derived from administrative health records, was used to exclude people who had physician-diagnosed diabetes (n = 839,127 in 2005) from the primary analyses. Diabetes tests included serum blood glucose (SBG), hemoglobin A1c (HbA1c), and oral glucose tolerance tests (OGTT).

**Results:**

In 2005, 37% of Ontario adults without pre-existing diabetes were tested with an SBG test, a 28% increase from 1995. The age-adjusted proportion of adults without diabetes undergoing a HbA1c test increased from 1.7% in 1995 to 6.0% in 2005. In 2005, a similar number of HbA1c tests were performed for individuals with diabetes (483,746) and without diabetes (496,616) despite large differences in the two groups' denominators. Less than 1% of Ontarians underwent OGTT testing in any year between 1995–2005. Nearly two-thirds of adults age 40 years and over had an SBG test over a 3-year period (April 1, 2002–March 31, 2005), in accordance with the Canadian Diabetes Association recommendations.

**Conclusion:**

Diabetes testing is common and has increased over the last ten years. Despite its absence in Canada's diabetes screening recommendations, HbA1c testing among individuals without diabetes is increasing rapidly, and OGTT, which is recommended, is rarely performed.

## Background

Diabetes and its complications are a significant cause of morbidity and mortality, and there is evidence to suggest an increasing burden of this disease both in Canada and globally [1,2]. Recent estimates from the province of Ontario suggest that 8.8% of Ontario adults have physician-diagnosed diabetes, which is up 69% from 1995 [3]. This increase has been attributed to a rise in type 2 diabetes (diabetes) cases, driven by an increase in obesity rates [4-8]. However, greater screening for diabetes over the last decade may have also contributed to these findings. Earlier studies have suggested that up to one-third of all 'true' diabetes cases are undiagnosed [9-12]. With increasing diabetes awareness and the recent publication of screening guidelines, the proportion of undiagnosed diabetes may have declined.

Because type 2 diabetes can remain asymptomatic for up to 10 years [13], in Canada two clinical guidelines recommend screening for the detection of diabetes in adults. Since 1998, the Canadian Diabetes Association (CDA) has recommended screening individuals aged 45 years and older every three years with a fasting blood glucose (FPG) test, and earlier and/or more frequently for individuals with risk factors [14]. In 2003, the CDA reduced the age to begin screening to age 40 and added a new recommendation for oral glucose tolerance testing (OGTT) for individuals with a FPG result of 5.7 to 6.9 mmol/L and at least one risk factor for diabetes [15]. However these recommendations were predominantly consensus-driven, due to insufficient evidence of direct benefits from screening (Grade D) [14,15]. The original CDA guidelines, published in 1992, did not include any screening recommendations outside of pregnancy [16]. The Canadian Task Force on Preventive Health Care [17] recommends screening only for adults with established hypertension or hyperlipidemia, for whom benefits of early diabetes detection and treatment have been shown.

We sought to explore trends in diabetes-related lab testing in Ontario from 1995 to 2005 in a descriptive analysis. Our first objective was to determine whether diabetes testing among Ontario adults without diabetes has increased over the last decade. Our second objective was to explore the extent to which the CDA screening guidelines are being followed in the province of Ontario, by examining how many Ontarians received blood glucose laboratory testing in accordance to the CDA guidelines. We chose to focus on the CDA guidelines because they affect a large population and they are increasingly referenced as a screening and testing strategy.

## Methods

### Data sources

Data were extracted from anonymised administrative health databases that include records for all individuals eligible for health services under the Ontario Health Insurance Plan (OHIP). All Ontario residents are eligible for OHIP coverage after 3 months of residency in the province. Legislation prohibits the private delivery of services covered under OHIP, including laboratory testing. The OHIP database was used to identify laboratory service claims for diabetes testing. We obtained demographic information from the Ontario Registered Persons Database (RPDB), and diabetes status from the Ontario Diabetes Database (ODD) [18]. These databases were linked anonymously using unique encrypted health card numbers.

Data from April 1, 1995 to March 31, 2006 were extracted for analysis. All data extraction and analyses were carried out using SAS (Version 9.1).

### Base population

A distinct base population was defined for each fiscal year under study (1995 to 2005), which was comprised of all Ontario adults aged 20 years or older recorded in the RPDB.

### Exclusion criteria

Persons with previously diagnosed diabetes (prevalent diabetes) were removed from each fiscal-year cohort. These individuals were identified using the ODD, a cumulative diabetes registry that uses administrative health records to determine diabetes status. Any individual having at least one hospital admission or two physician claims bearing a diabetes diagnosis within two years is defined as having physician-diagnosed diabetes and is included in the database. Women with gestational diabetes are excluded from the registry. The ODD has been validated using primary health care data, and shown to have a sensitivity of 86% and a specificity of 97%. A detailed description of its methodology can be found elsewhere [18]. Incident cases of diabetes were not excluded.

### Determination of diabetes-related laboratory tests

Ontario adults without diabetes who underwent diabetes-related laboratory tests were identified using OHIP claims bearing their encrypted health card numbers. The OHIP database records all laboratory tests performed outside of hospitals. The diabetes-related tests of interest were serum blood glucose (SBG) (codes G002, L111, L112), hemoglobin A1c (HbA1c) (code L093), and oral glucose tolerance test (OGTT) (code L104) (17). If an individual had more than one SBG record (code L111) in the same day, this was considered to represent one OGTT rather than two SBG tests. Glucose tolerance tests in pregnancy have their own unique fee code and were excluded from our analyses. The SBG code does not discriminate between fasting and random serum blood glucose measurements. Records for the laboratory fee code of L700, a patient documentation and specimen collection fee submitted for the processing of any lab test in Ontario, were also collected as a measure of general laboratory testing. Only one L700 claim can be submitted per patient per day, regardless of how many tests are carried out for an individual [19]. OHIP records for HbA1c and OGTT testing in 2005 were also reviewed for individuals with diagnosed diabetes.

### Percentage of Ontarians tested per fiscal year

For each laboratory test, the percentage of Ontarians aged 20 and older tested was determined for each fiscal year from 1995 to 2005. The numerator was the number of individuals who underwent at least one test of interest that year. For each test, individuals contributed equally whether they had one or multiple tests within each year. The denominator for each year was the number of Ontario adults aged 20 years or older (based on Statistics Canada Census population counts), minus the number of prevalent diabetes cases derived from the ODD. Estimates of the Ontario population provided by Statistics Canada were used for years in between Census sampling. To adjust for differences in the age structure of the Ontario population over time, direct age standardization using the 1991 Canadian Census population was performed.

### Sensitivity analysis

For people newly diagnosed with diabetes, our dataset provided only the year in which diabetes was diagnosed. Thus, for lab tests performed during the year a person was newly diagnosed with diabetes, we could not determine whether their lab tests were ordered before (for screening and diagnosis) or after the establishment of a diabetes diagnosis. To investigate the extent to which this may have influenced our results, a sensitivity analysis was performed in which we excluded all lab tests carried out in individuals diagnosed with diabetes in the same year (incident cases).

### Percentage of Ontarians over age 40 tested over one to five years

To estimate the number of Ontarians who were screened in accordance with the CDA guidelines, we first identified a cohort of Ontarians who were free of diabetes on April 1^st ^2005. Next, we examined the percentage of people tested in the most recent year (April 1, 2004 to March 31, 2005). Next, the percentage of people who were tested over 2, 3, 4 and 5 year periods was estimated by the corresponding historic data (e.g. the 5 year period was April 1, 2000 to March 31, 2005). Only Ontarians age 40 years or older for the entire 5-year period were included in these estimates.

### Research ethics

Ethics approval was obtained from the institutional review board at Sunnybrook Health Sciences Complex, Toronto, Ontario, Canada.

## Results

**Figure 1:**
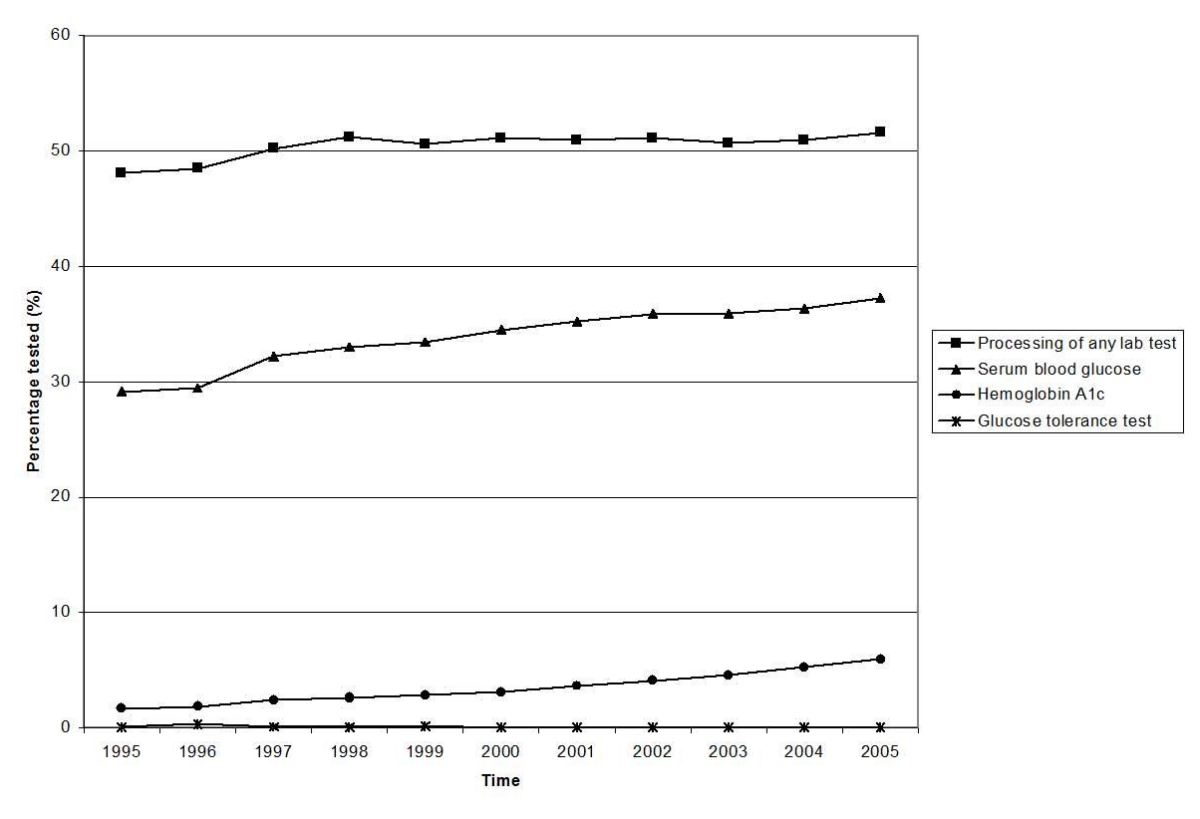
**Percentage of Ontario adults (20 and older) tested with diabetes-related investigations 1995–2005 (Age-standardized using 1991 Canadian Census population)**.

Both diabetes-related and general laboratory tests are commonly performed (Figure 1 and Table 1). SBG and HbA1c testing have increased steadily since 1995, despite a relative plateau in the rate of laboratory testing in general (approximately 50% of Ontarians received at least one lab test yearly). In 2005, the age-adjusted percentage of Ontario adults without diabetes undergoing an SBG test was 37%, representing a 28% increase since 1995.

**Table 1:** Un-standardized number of diabetes-related lab tests among Ontario adults (20 and older) without an established diagnosis of diabetes in the preceding fiscal year 1995–2005.

Year	Processing of any lab test	Serum blood glucose	HbA1c	OGTT
2005	4,512,614 (70,020)	3,301,311 (67,299)	496,616 (54,706)	2,452 (285)
2004	4,404,670 (71,258)	3,180,760 (68,675)	464,471 (54,040)	2,188 (254)
2003	4,319,405 (64,986)	3,093,533 (62,661)	398,624 (47,536)	1,430 (151)
2002	4,294,975 (65,392)	3,040,925 (62,801)	350,966 (46,843)	1,357 (163)
2001	4,204,571 (62,655)	2,931,753 (59,851)	305,803 (43,154)	1,352 (210)
2000	4,142,494 (55,488)	2,819,595 (52,663)	261,396 (37,352)	1,338 (154)
1999	4,039,243 (53,371)	2,691,883 (50,784)	228,395 (35,678)	11,200 (492)
1998	4,037,268 (50,665)	2,621,334 (48,158)	207,735 (32,330)	5,803 (1,006)
1997	3,917,028 (49,002)	2,524,294 (46,734)	188,019 (30,488)	6,640 (1,327)
1996	3,731,845 (43,963)	2,274,260 (41,707)	144,266 (24,843)	25,082 (1,982)
1995	3,663,118 (43,100)	2,222,656 (41,321)	126,192 (22,747)	5,944 (1,303)

The number of tests conducted in newly diagnosed diabetes cases (incident cases in the year under study) is expressed in parentheses.

The most striking increase in diabetes-related testing was observed for HbA1c tests, which increased approximately 10% per year or 250% from 1995 to 2005 (Figure 1). The age-adjusted proportion of adults without diabetes who underwent the test in 2005 was 6.0%, compared to 1.7% in 1995. In 2005, 8.4% of adults aged 40 years and older, without diabetes underwent a HbA1c test (results not shown). In 2005, HbA1c was performed for 496,616 individuals without diabetes, and for 483,746 individuals with diagnosed diabetes. An additional 54,706 tests were completed amongst individuals newly diagnosed with diabetes in 2005 bringing the total number of diabetes patients tested to 538,452 (64% of diabetics). The OGTT continues to be underused with less than 1% of adults being tested, and its use has decreased 60% from 0.08% in 1995 to 0.03% in 2005 (Figure 1). In 2005, 3,241 individuals underwent at least one OGTT. Among the individuals who underwent an OGTT in 2005, 2,452 did not have diabetes, 504 had a pre-existing diagnosis of diabetes, and 285 were newly diagnosed with diabetes in 2005. The results of the sensitivity analysis demonstrate that these broad trends in diabetes laboratory testing still hold, even if all tests conducted among incident cases of diabetes are removed from the analysis (Table 1).

Table 2 documents the percentage and number of people age 40 or older who received a SBG test increased when more than one year of historical data was examined. 40% of people were tested within one year (2005), 63% by three years (the screening interval recommended by CDA), and by five years, 71% had undergone at least one SBG test.

**Table 2:** Serum blood glucose testing in a cohort of Ontario adults aged 40 years and older, without diabetes, 2001–2005.

Time period	Number new tests	Denominator*	% newly tested	Cumulative no. tested	Denominator (total)	Cumulative % tested	Never tested
1 year (2005)	2,043,156	5,071,304	40.30%	2,043,156	5,071,304	40.30%	
2 years (2004–2005)	766,250	3,028,148	25.30%	2,809,406	5,071,304	55.40%	
3 years (2003–2005)	374,702	2,261,898	16.60%	3,184,108	5,071,304	62.80%	
4 years (2002–2005)	244,953	1,887,196	13.00%	3,429,061	5,071,304	67.60%	
5 years (2001–2005)	185,139	1,642,243	11.30%	3,614,200	5,071,304	71.30%	28.70%

* Denominator refers to the number of individuals in the cohort who had not previously undergone a serum blood glucose test during the study period (2001–2005)

## Discussion

This study documents a 28% increase in serum glucose testing between 1995 and 2005 among Ontarians without diabetes. People who advocate for screening may find the current level of testing unacceptably low. Others may be encouraged that the level of testing (two thirds of Ontarians recommended to be screened by the CDA are screened) is similar to other well-established programs such as mammography [20,21] and papanicolaou (Pap) testing [22,23], despite the absence of a formal diabetes screening program. Still others may find the level of testing too high, given the more conservative recommendations of the Canadian Task Force on Preventive Health Care and the grade D level of evidence of the CDA recommendations.

OGTT testing in Ontario is very uncommon and has declined over the last 10 years. Lyon et al. [24] showed 14.8% of residents in the Canadian city of Calgary tested with a serum glucose fell into the range of 5.7 and 6.9 mmol/L and would have required an OGTT based on the CDA guidelines. They noted that if these were all followed by an OGTT, as recommended by the CDA, it would result in an additional 2823 OGTTs per month, 19 times greater that their baseline rate of 150 tests per month (excluding tests in pregnant women). In Ontario, 0.03% of the total population (3,241 individuals with and without diabetes) received an OGTT in 2005. This number likely over-estimates the true number of OGTT carried out in Ontario as our study methodology counted any individual who underwent two SBG tests in a single day as undergoing an OGTT. These individuals may in fact represent individuals presenting for fasting and postprandial blood glucose measurements. However, this bias towards over-estimation of OGTT further reinforces our finding that this test is infrequently used in Ontario. The CDA recommendations for OGTT are clearly unpopular with clinicians and/or patients. For example, the ADDITION trial of population-based screening for diabetes in the Netherlands found a dropout rate of 23% among participants advised to undergo an OGTT as part of the screening algorithm [25]. The infrequent use of the OGTT in Ontario is likely to have important consequences with respect to diabetes case detection. Using only fasting plasma glucose will fail to diagnose approximately 30% of individuals with diabetes [26,27], and an even greater proportion among the elderly [28].

Conversely, our study found a 250% increase in HbA1c testing among persons without diabetes, a test that has not been recommended for diabetes screening due to concerns regarding reliability [29]. If this rate of increase is sustained, the number of individuals without diabetes who undergo HbA1c testing each year may soon exceed the number of patients with diabetes tested. These data suggest that some clinicians may be using the HbA1c test as an alternative to the recommended FPG and OGTT to diagnose diabetes. Clinicians and patients may prefer the HbA1c test, as there is no requirement for fasting or multiple blood draws, there is a threshold level associated with retinopathy and cardiovascular mortality [29], and it can be used to guide treatment decisions in the management of diabetes. A recent systematic review [30] concluded that the HbA1c and FPG are equally effective screening tools for the detection of diabetes. The combined use of HbA1c and FPG has been shown to have greater sensitivity than the use of FPG alone [31] in diabetes diagnosis but the impact of additional HbA1c testing in Ontario on diabetes case detection is uncertain, particularly in light of the low uptake of the OGTT.

The strengths of our study include the use of population-based, individually linked administrative data and the use of a validated diabetes database to exclude diabetes patients [18]. However, there are some important limitations that merit emphasis. First, we were not able to differentiate between fasting and random serum blood glucose tests using our data. Since only fasting blood glucose testing is recommended for diabetes screening, we could not determine which tests were ordered specifically for the purpose of asymptomatic diabetes screening versus case finding in symptomatic persons. Second, diabetes cases were excluded based on a registry that is associated with a 14% false negative rate, therefore some persons with diabetes may have been misclassified and included in our study. Third, although we have captured all diabetes-related laboratory services billed to OHIP, we were not able to capture tests carried out within hospital in-patients. However this limitation at most resulted in an underestimate of our findings.

## Conclusion

In summary, diabetes-related lab testing is common in Ontario and has increased substantially from 1995 to 2005. HbA1c testing for people without diabetes is commonly performed, but not recommended – while OGTT is recommended but rarely performed. Regardless, the majority of Ontarians are likely being tested for diabetes, despite the controversy regarding diabetes screening and the lack of a formal screening program. The rise in testing for diabetes possibly, if not likely, contributed to the overall increase in diabetes observed during this time period [3]. Whether the increased testing resulted in a change in the ratio between diagnosed and undiagnosed cases of diabetes is uncertain but should be addressed in future studies which explore the socio-demographic characteristics and co-morbidities of those who undergo diabetes testing.

## Abbreviations

CDA: Canadian Diabetes Association; FPG: Fasting plasma glucose; HbA1c: Hemoglobin A1c; ODD: Ontario Diabetes Database; OGTT: Oral glucose tolerance test; OHIP: Ontario Health Insurance Plan; RPDB: Registered Persons Database; SBG: Serum blood glucose.

## Competing interests

The authors declare that they have no competing interests.

## Authors' contributions

SEW and DGM formulated the study's design. SEW was responsible for data acquisition and drafted the original manuscript. SEW, LLL, LCR, and DGM made substantial contributions to the analysis and interpretation of data and revised the manuscript for important intellectual content. All authors read and approved the final manuscript.

## Pre-publication history

The pre-publication history for this paper can be accessed here:


